# Supplementary material for: The association between prolonged SARS-CoV-2 symptoms and work outcomes
Source: PLoS One. 2024 Jul 29;19(7):e0300947. doi: 10.1371/journal.pone.0300947 (PMC11285965; doi:10.1371/journal.pone.0300947)
Supplement: S1 Appendix — (DOCX) [file pone.0300947.s001.docx]

**INSPIRE Group**

**Rush University, Administrative Core & Enrolling Site**

**Core research team:** Michelle Santangelo, MS, Research Manager; Katherine Koo, MS-HSM, Program Manager; Antonia Derden, BA, Administrative Assistant

**Site research team:** Kristyn Gatling, MA, Research Coordinator. Research Assistants: Diego Guzman, BS; Geoffrey Yang, BA; Amro (Marshall) Kaadan, BS; Minna Hassaballa, BA; Ryan Jerger; Zohaib Ahmed, BS; Michael Choi, MBS, BA; Ariana Pavlopoulos, BS; Avinash Kesari, BS; Caitlin A Gaylord; Chloe Gomez; Elizabeth Lomas, BS; Phouthavang (Jimmie) Boliboun, BS; Krisna Patel, BS

**Yale University, Analytic Core & Enrolling Site**

**Core research team:** Caitlin Malicki, MPH, Senior Research Manager. Core Statisticians: Zhenqiu Lin, PhD; Shu-Xia Li, PhD; Huihui Yu, PhD; Imtiaz Ebna Mannan, MS; Zimo Yang, MS; Mengni Liu, MS

**Site Investigators:** Andrew Ulrich, MD, Co-Investigator

**Site research team:** Jeremiah Kinsman, MPH, NREMT, Research Manager; Jocelyn Dorney, MPH, Research Coordinator. Research Assistants: Zihan Diao, Senyte Pierce; Xavier Puente; Wafa Salah

**University of Washington, Clinical Core & Enrolling Site**

**Core Investigators:** Graham Nichol, MD, Principal Investigator;

**Core research team:** Jill Anderson, BSN, RN, Clinical Core Program Manager; Mary Schiffgens, Grant & Finance Manager; Dana Morse, RN, BSN, Research Coordinator; Karen Adams, BA, Regulatory Specialist; Tracy Stober, BA, MA, Patient Representative; Zenoura Maat, Research Assistant

**Site Investigators:** Nikki Gentile, MD, PhD, Co-Investigator

**Site research team:** Research Coordinators: Rachel E. Geyer, MPH; Michael Willis, AS, BSHS; Zihan Zhang, Analyst; Gary Chang, PhD, Senior Biostatistician. Victoria Lyon, MPH, Project Manager. Research Assistants: Robin E. Klabbers, MSc in Medicine, MSc in Global Health; Luis Ruiz, BA; Kerry Malone, BA; Jasmine Park

**Thomas Jefferson University, Enrolling Site**

**Site Investigators:** Efrat Kean, MD, Co-Investigator

**Site research team: Nurse Coordinator:** Nicole Renzi, RN. Program Manager: Phillip Watts, BA, MM, CCRP. Research Coordinators: Morgan Kelly, BS; Kevin Schaeffer, BS; Dylan Grau, BS; ; David Cheng, BS; Carly Shutty, BSN; Alex Charlton, BS; Lindsey Shughart, BS; Hailey Shughart, BA, CCRP; Grace Amadio, MD, CCRP; Jessica Miao, BA. Research Assistant: Paavali Hannikainen, BS.

**University of California, Los Angeles, Enrolling Site**

**Site Investigators:** Lauren E. Wisk, PhD, Co-Investigator

**Site research team:** Michelle L’Hommedieu, PhD, Site Program Director; Chris Chandler, BA, Research Assistant; Megan Eguchi, MPH, Data Analyst; Kate Diaz Roldan, MPH, Research Assistant; Raul Moreno, BA, Administrative Analyst

**University of California, San Francisco, Enrolling Site**

**Site Investigators:** Ralph C. Wang, MD, MAS, Site Principal Investigator;

**Site research team:** Robin Kemball, MPH, Program Manager; Research Coordinators: Virginia Chan, MPH; Cecilia Lara Chavez; Angela Wong, BA; Mireya Arreguin

**University of Texas Health Science Center at Houston, Enrolling Site**

**Site Investigators:** Ryan Huebinger Site, MD, Site Principal Investigator.

**Site research team**: Arun Kane, BA, Research Coordinator; Peter Nikonowicz, BA, Research Coordinator; Sarah Sapp, MPH, Research Coordinator

**University of Texas Southwestern Medical Center, Enrolling Site**

**Site Investigators:** Samuel McDonald, MD, Co-Investigator

**Site research team:** David Gallegos, Research Coordinator; Riley Martin, Research Assistant

**Centers for Disease Control and Prevention (CDC)**

**Investigators:** Sharon Saydah, PhD; Ian D. Plumb, MBBS, MSc; Aron J. Hall, DVM, MSPH; Melissa Briggs-Hagen, MD, MPH
