# Supplementary material for: The association between prolonged SARS-CoV-2 symptoms and work outcomes
Source: PLoS One. 2024 Jul 29;19(7):e0300947. doi: 10.1371/journal.pone.0300947 (PMC11285965; doi:10.1371/journal.pone.0300947)
Supplement: Attachment — Submitted filename: Response to Reviewers.docx [file pone.0300947.s002.docx]

June 14, 2024

Dear PLOS ONE Editorial Board,

We wish to thank you and the reviewers for your insightful comments regarding our manuscript, “**The Association Between Prolonged SARS-CoV-2 Symptoms and Work Outcomes**”. Your suggestions have greatly helped us to improve the quality of our analysis and findings. We have made many significant revisions, including the addition of three new figures/tables and relevant updates to the methods, results and discussion sections of the manuscript. We have also revised our data sharing policy and confirm that the following statement is true regarding competing interests: “These do not alter our adherence to PLOS ONE policies on sharing data and materials.”

Please find a point-by-point response to reviewers following this letter. We hope that these revisions are sufficient to make our manuscript suitable for publication in the PLOS ONE and look forward to hearing from you at your earliest convenience.

Sincerely,

Arjun Venkatesh, MD, MBA, MHS
Professor of Emergency Medicine
Chief, Department of Emergency Medicine
Yale University School of Medicine
Chief, Emergency Services
Yale New Haven Hospital

Response to Reviewers

Administrative Revisions:
**1. Please ensure that your manuscript meets PLOS ONE's style requirements, including those for file naming**.

We have updated our files and file names per guidelines.

**2. We note that one or more of the authors have an affiliation to the commercial funders of this research study: University of Texas Southwestern Medical Center. 2a. Please provide an amended Funding Statement declaring this commercial affiliation, as well as a statement regarding the Role of Funders in your study.** **2b. Please also provide an updated Competing Interests Statement declaring this commercial affiliation along with any other relevant declarations relating to employment, consultancy, patents, products in development, or marketed products, etc.**  

We have reviewed the commercial funding with the relevant author (AHI) and they confirmed that the funding organization did not play a role in the study design, data collection and analysis, decision to publish, or preparation of the manuscript and also did not provide financial support in the form of authors' salaries. We attempted to update the conflicts of interest via the submission portal but were unable to do so. As such, we ask that you remove this declaration and update our competing interests as follows:

""I have read the journal's policy and the authors of this manuscript have the following competing interests:

AHI receives research grant funding from the University of Texas Southwestern Medical Center outside of the submitted work.

AKV receives grants from the Agency for Healthcare Research and Quality and the SAEM Foundation outside the submitted work.

ESS receives grant funding from the National Heart, Lung, and Blood Institute (R01HL151240), and the Patient Centered Outcomes Research Institute (HM-2022C2-28354).

JCCM receives research grant funding from SAMHSA (1H79TI084428-01 and 1H79TI085981-01, PI LeSaint), FDA (75F40122C00116, PI Anderson), NIH-NINDS (U24NS129501, PI Rodriguez) outside the submitted work.

JE is Editor-in-chief of the Adult Primary Care topics at UpToDate.

KLR receives research grant funding from Abbott Diagnostics, DermTech, MeMed, Prenosis, Siemens Healthcare Diagnostics, PROCOVAXED funded by NIAID 1R01AI166967, and PREVENT funded by CDC U01CK00048 outside the submitted work.

KNO receives research grant funding for PROCOVAXED funded by NIAID R01 AI166967, PI: Rodriguez outside the submitted work.

MG receives grant funding from the Biomedical Advanced Research and Development Authority Research Grant, the Bill and Melinda Gates Foundation, and the Society of Directors of Research in Medical Education Grant outside the submitted work.

MJH receives research grant funding from an Investigator Award from Merck, MISP 100099, PI: Hill outside the submitted work.

The following authors have declared that no competing interests exist: HY, KAS, RAW."

**3. Please confirm that Competing Interests do not alter your adherence to all PLOS ONE policies on sharing data and materials.**

We have noted the following statement in our cover letter, which you have advised you will change on our behalf. "This does not alter our adherence to PLOS ONE policies on sharing data and materials.”

**4. We note that you have indicated that there are restrictions to data sharing for this study. If there are ethical or legal restrictions on sharing a de-identified data set, please explain them in detail**

Study data is owned and managed directly by the grant recipient (Rush University) and the funder (Centers for Disease Control and Prevention). We ask that you update the data availability statement as follows: The data underlying the results presented in the study are from the INSPIRE Registry. The coordinating center, Rush University, can be contacted via email at inspirepub@rush.edu to request information related to confidential data.

**5.  One of the noted authors is a group or consortium "INSPIRE Group". In addition to naming the author group, please list the individual authors and affiliations within this group in the acknowledgments section of your manuscript. Please also indicate clearly a lead author for this group along with a contact email address.**

We have noted on the title page that membership of the INSPIRE Group is provided in the S1 Appendix. The appendix lists all individual authors and their affiliations.

**6. Please include your full ethics statement in the ‘Methods’ section of your manuscript file. In your statement, please include the full name of the IRB or ethics committee who approved or waived your study, as well as whether or not you obtained informed written or verbal consent.**

We have updated the “Methods” section of our manuscript accordingly.

Overall Reviewer Comments:

**1. The study enrolled participants from eight different sites, but the data related to these sites has not been included in the table based on the number of symptoms. As such, it is unclear if the authors adjusted their model to account for any variation between these sites. It would be helpful to know the strategies the authors used to ensure that the results are not biased by differences between the sites.**

Study enrollment occurred from 8 sites. However, because the study was conducted in a de-centralized fashion, anyone could enroll in the study across the United States. As a result, site-specific effects may be less pronounced than anticipated. Also, in prior publications from the INSPIRE registry, we have acknowledged that select demographic differences between sites supported the efforts to recruit a more diverse population. However, that population may differ from the general U.S. population as shown in table 1.

**2. One of the reviewers pointed out that using hair loss as an indicator of asymptomatic COVID-19 cases is not accurate. This, along with the classification of the number of symptoms category, raises questions about the validity of the analysis. The authors should consider alternative measures to accurately identify asymptomatic cases and reclassify the number of symptoms categories to ensure that the analysis is valid.**

We considered hair loss to be a symptom of SARS-CoV-2 and did not consider it as an indicator of asymptomatic cases. We defined asymptomatic as responding to "none of the above" to the symptom screener.

We measured symptoms utilizing the Centers for Disease Control and Prevention “Persons Under Investigation” (PUI) symptom list for SARS-CoV-2, which is a standard tool that has been the basis for multiple prior publications from the INSPIRE registry and is detailed in the study protocol (O'Laughlin KN, et al. Study protocol for the Innovative Support for Patients with SARS-COV-2 Infections Registry (INSPIRE): A longitudinal study of the medium and long-term sequelae of SARS-CoV-2 infection. PLoS One. 2022 Mar 3;17(3):e0264260. doi: 10.1371/journal.pone.0264260). Use of this symptom screener for research purposes is also further supported by 6 INSPIRE peer-reviewed publications in distinct journals using these definitions.

**3. The study data showed that recall bias occurred, leading to biased estimates of associations between exposures and outcomes. It is important that the authors implement strategies to minimize the recall bias and enhance the validity and reliability of study findings. The authors should consider using objective measures, such as biomarkers or medical records, to validate self-reported data. Additionally, they should consider conducting a sensitivity analysis to determine the impact of recall bias on the study's findings.**

As we restricted our study sample to verified COVID+ participants and to 3-month outcomes, we utilized data for this analysis with the shortest possible time-window between acute COVID illness and outcome ascertainment to minimize any recall bas. As such, any sensitivity analysis utilizing longer term outcome data would be potentially more prone to recall bias.

Reviewer 1 comments:

**1. Vaccination status was included in the participant characteristics. Did the authors include the FDA-approved inhibitor, Paxlovid? That inhibitor was used as an antiviral.**

We did not include Paxlovid or other medication for COVID-19 treatment in the risk adjustment model as this data was not universally available and because the association between each drug that has been approved for COVID-19 and prolonged symptoms or work effects is still speculative at best.

2. **The questions in the survey seem need to differentiate the mild symptoms and the severe symptoms as different symptoms have different effect. Simply count the numbers of symptoms may provide misleading results. What is the rational to include hair loss as one of the symptoms?**

Symptoms were collected using the Centers for Disease Control and Prevention “Persons Under Investigation” (PUI) symptom list for SARS-CoV-2, which is a standard tool that has been the basis for multiple prior publications from the INSPIRE registry and is detailed in the study protocol (O'Laughlin KN, et al. Study protocol for the Innovative Support for Patients with SARS-COV-2 Infections Registry (INSPIRE): A longitudinal study of the medium and long-term sequelae of SARS-CoV-2 infection. PLoS One. 2022 Mar 3;17(3):e0264260. doi: 10.1371/journal.pone.0264260). We have clarified this in the methods section of the paper. Unfortunately, symptoms were not differentiated between mild, moderate and severe within this validated tool for symptoms. We have acknowledged this as a limitation in prior studies of prolonged symptoms and the following statement is included in the limitations section of this paper: “Second, our analyses considered each symptom equally, which may not capture nuanced relationships between symptom type, symptom severity and work outcomes.”

3. **Participants enrolled into the survey are from eight universities in US. Participants from companies may also needed.**

We did not have any industry or “company” based enrollment or participation. Furthermore, the enrolled participants were not from universities but rather could be enrolled across the U.S. by any of the eight U.S. participating sites, many of which conducted recruitment in partnership with local public health agencies or through virtual platforms to ensure a diverse recruitment population.

Reviewer 2 comments: 

**1. Line 126. It appears that the COVID-19 symptoms were self-reported by the participants. How did you mitigate recall bias, especially for COVID-19 symptoms that are easily confused with other conditions? If recall bias is acknowledged, it should be discussed in the limitations section of the manuscript.**

We believe the risk of recall bias is limited for several reasons. First, the actual survey question stems and structure include multiple references and guides toward COVID-19 illness as described in reference 13. We have updated the discussion to reflect this. In addition, this analysis was limited to COVID+ participants and the study title and purpose was to study long-term outcomes of COVID-19 creating a study frame that disproportionately is focused on COVID-19.

**2. Line 146: Regarding the questions asked of participants, such as "COVID-19-like symptoms," how can participants without a medical background accurately identify COVID-19-like symptoms? Do you provide a list of symptoms, or is medical proof required from participants?**

All participants were given structured question via web-based surveys that included standard symptom lists derived from the CDC Persons Under Investigation list referenced above. As aforementioned, this symptom list has been utilized in prior studies from our CDC-funded registry as well as other studies to examine COVID-19.

**3. Regarding the surveys, did you include questions about mental health disorders following COVID-19? Depression or anxiety could also prevent patients from returning to work, making it a relevant factor to consider in your study.**

Our registry includes the ascertainment of several mental health outcomes via the PROMIS-29 v2.1 administered in the INSPIRE study. Outcomes measured in the PROMIS-29 assessment, such as anxiety, depression, and the ability to participate on social roles and activities, have been included in the following publication and were not included in this focused analysis of work outcomes. Wisk LE, Gottlieb MA, Spatz ES, et al. Association of Initial SARS-CoV-2 Test Positivity With Patient-Reported Well-being 3 Months After a Symptomatic Illness. JAMA Netw Open. 2022;5(12):e2244486. doi:10.1001/jamanetworkopen.2022.44486

**4. Concerning the analysis results, did you investigate any potential interaction effects? Additionally, could you stratify the results based on high, middle, and low income levels? This would help determine whether the impact of COVID-19 symptoms to work varies across different income groups, which could provide more nuanced and informative insights.**

Thank you for your suggestion. We have now stratified work loss by income level in Table 2, the findings of this exploratory analysis and implications of which have been incorporated into the results and discussion.

**5. Which symptoms were most (or top 5?) significantly associated with affecting work outcomes? Additionally, did you find that vaccination had a protective effect that helped patients return to work earlier? These insights could be informative, and they would add considerable value to your study.**

Thank you for your question. We have added two new figures to our paper to compare work outcomes (missed workdays and return to work) by symptom number and type. Findings and implications of this secondary analysis were added to the Results and Discussion section of the paper. We did not, however, direct any specific analysis towards vaccination because our cohort was disproportionately vaccinated as evidenced in Table 1 and would likely not provide a meaningful comparison to unvaccinated individuals.

**6. In the discussion, it would be beneficial to explore the possible mechanisms or pathways through which Long COVID may affect work outcomes. One suggestion is to consider discussing the findings from recent retrospective cohort studies on Long COVID effects. For example, COVID-19 may increase the risk of different kind of health outcomes (Bowe, B., Xie, Y. & Al-Aly, Z. Postacute sequelae of COVID-19 at 2 years. Nat Med 29, 2347–2357 (2023). https://doi.org/10.1038/s41591-023-02521-2). Long COVID may increase the risk of depression and anxiety. (Zhang Y, Chinchilli VM, Ssentongo P, et al Association of Long COVID with mental health disorders: a retrospective cohort study using real-world data from the USABMJ Open 2024;14:e079267. doi: 10.1136/bmjopen-2023-079267). Post-hospitalization COVID-19 may lead to disabilities and financial problems. (Admon AJ, Iwashyna TJ, Kamphuis LA, et al. Assessment of Symptom, Disability, and Financial Trajectories in Patients Hospitalized for COVID-19 at 6 Months. JAMA Netw Open. 2023;6(2):e2255795. doi:10.1001/jamanetworkopen.2022.55795). Discussing these aspects and more references could make your discussion more comprehensive.**

We appreciate this input. We have incorporated all the above references in the discussion and expanded the discussion to include more hypotheses regarding the potential mechanism between prolonged symptoms of COVID-19 and work outcomes.
